# The Interplay of Adipokines, Body Composition and Glucose Homeostasis in Pregnant Women with a History of RYGB Operation

**DOI:** 10.3390/nu15112498

**Published:** 2023-05-27

**Authors:** Luise Bellach, Liliana-Imi Gard, Simon David Lindner, Sabina Baumgartner-Parzer, Peter Klimek, Alexandra Kautzky-Willer, Michael Leutner

**Affiliations:** 1 Clinical Division of Endocrinology and Metabolism, Department of Internal Medicine III, Medical University of Vienna, Spitalgasse 23, A-1090 Vienna, Austria; 2 Section for Science of Complex Systems, CeMSIIS, Medical University of Vienna, Spitalgasse 23, A-1090 Vienna, Austria; 3 Complexity Science Hub Vienna, Josefstädter Strasse 39, A-1080 Vienna, Austria

**Keywords:** bariatric surgery, pregnancy, adipokines, bioelectrical impedance, glucose control

## Abstract

Roux-en-Y gastric bypass operations (RYGB-OP) and pregnancy alter glucose homeostasis and the adipokine profile. This study investigates the relationship between adipokines and glucose metabolism during pregnancy post-RYGB-OP. (1) Methods: This is a post hoc analysis of a prospective cohort study during pregnancy in 25 women with an RYGB-OP (RY), 19 women with obesity (OB), and 19 normal-weight (NW) controls. Bioimpedance analysis (BIA) was used for metabolic characterization. Plasma levels of adiponectin, leptin, fibroblast-growth-factor 21 (FGF21), adipocyte fatty acid binding protein (AFABP), afamin, and secretagogin were obtained. (2) Results: The phase angle (φ) was lower in RY compared to OB and NW. Compared to OB, RY, and NW had lower leptin and AFABP levels, and higher adiponectin levels. φ correlated positively with leptin in RY (R = 0.63, *p* < 0.05) and negatively with adiponectin in OB and NW (R = −0.69, R = −0.69, *p* < 0.05). In RY, the Matsuda index correlated positively with FGF21 (R = 0.55, *p* < 0.05) and negatively with leptin (R = −0.5, *p* < 0.05). In OB, FGF21 correlated negatively with the disposition index (R = −0.66, *p* < 0.05). (3) Conclusions: The leptin, adiponectin, and AFABP levels differ between RY, OB, and NW and correlate with glucose metabolism and body composition. Thus, adipokines might influence energy homeostasis and maintenance of cellular health during pregnancy.

## 1. Introduction

Obesity is an ever-increasing issue in industrialized societies. In fact, according to the World Health Organization, up to 13% of the world’s population suffered from obesity in 2016 (women more often than men [1]). Bariatric surgery poses a viable and potent treatment option, vastly improving cardiometabolic outcomes. In the case of the Roux-en-Y gastric bypass (RYGB), one of the most common bariatric operation techniques [2], the undigested chymus bypasses the duodenum, thus directly stimulating the ileal cells to secrete glucagon-like-peptide 1 (GLP−1), leading to a drastically altered serum profile of gut peptides and, in further consequence, insulin [3]. In the long run, this leads to a remarkable improvement in glucose homeostasis, as can be seen by the return to euglycemia in over 80% of patients with dysglycemia [4], but also the risk of hypoglycemic events can be as high as 50% [5]. An exaggerated risk can also be seen in pregnant women with a history of RYGB [6], albeit that pregnancy per se increases the risk of developing a transient state of insulin resistance in a non-bariatric cohort, especially in the second and third trimesters. If additional risk factors such as obesity or positive family history are present, gestational diabetes mellitus (GDM) might develop [7]. GDM is a common disease in pregnancies of multifactorial origin, however, changes in gestational hormone levels have been shown to modulate insulin sensitivity according to the pregnancy stage. Another potential risk factor GDM might pose is the dysbalances in adipokine levels (hormones derived from the adipose tissue which has, next to energy storing and insulating properties, also endocrine functions [8]). As, due to the accentuated weight loss, adipose tissue recedes after a RYGB operation, alterations in adipokine have been observed [9]. Adipokines, such as leptin, adiponectin, and fibroblast growth factor 21 (FGF21), are known to play a central role in energy homeostasis on a more systemic level. They regulate appetite, glucose, and lipid metabolism and can act both by stimulating and downregulating the immune system [10,11] and are thus key players in mediating cardiometabolic diseases. Known for its appetite-suppressing and pro-inflammatory effects, leptin also influences insulin resistance depending on the abundance of leptin levels. This stands in contrast to adiponectin which acts as an anti-inflammatory agent and has insulin-sensitizing properties. Obesity is linked to high leptin, yet low adiponectin levels [12]. Moreover, in recent years, interest has spiked around an adipocyte fatty acid-binding protein (AFABP) due to its role in metabolic inflammation [13], the liver-derived glycoprotein afamin due to its connection with the metabolic syndrome [14], and secretagogin due to its relation to insulin secretion [15]. Interestingly, in pregnancy, the elevation of AFABP and leptin, as well as decreased adiponectin, have been implicated in the development of GDM. Secretagogin is related to the regulation of insulin secretion [16], and elevated afamin levels have been associated with adverse pregnancy outcomes such as preeclampsia [14]. As RYGB also is inherently connected to both glucose metabolism and the alteration of the adipokine pool [3,9], it remains to be determined how these factors interact with each other during pregnancy. Thus, this study sets out to assess the implications of changes in the different adipokines for the glucose metabolism in pregnant women with a history of RYGB operation compared to women with obesity and normal-weight controls.

## 2. Materials and Methods

A detailed description of the study methods can be found in earlier publications [6,17].

### 2.1. Design

This is a post hoc analysis of a prospective cohort study conducted at the Medical University of Vienna and is set out to assess the differences and changes regarding glucose and lipid metabolism in pregnant women with an RYGB compared to pregnant women with obesity, but without an RYGB, and normal-weight pregnant controls. The study has been conducted in accordance with the declaration of Helsinki and has been approved by the Ethics Committee of the Medical University of Vienna (ethics referral number 1364/2022). Informed consent was obtained prior to enrollment into this observational study.

### 2.2. Study Population

The study population consists of 25 pregnant women with an RYGB and a median time span between operation and pregnancy of 3.3 years, 19 pregnant women with obesity (BMI ≥ 35 kg/m^2^) but without a history of bariatric surgery, and 19 normal-weight (BMI < 25 kg/m^2^) pregnant women. Exclusion criteria were bariatric surgery other than RYGB, the presence of infectious diseases, and liver or renal disease.

### 2.3. Tests

The oral glucose tolerance test (OGTT) was conducted between the 24th and the 28th week of gestation as well as 3 to 6 months post-partum. After an overnight fast, a baseline blood sample was drawn and 75 g of glucose was taken up orally, followed by repeated measurements every 30 min after ingestion. Within 7 to 14 days of the OGTT, an intravenous glucose tolerance test (IVGTT) was performed. Upon sampling of a baseline measurement, 0.3 g/kg of glucose was infused intravenously. After 20 min, 0.05 IU/kg body weight of insulin was infused, followed by regular measurements of blood glucose, insulin, and C-peptide levels up to 60 min after initiation of the IVGTT. Blood glucose levels below 54 mg/dL were defined as hypoglycemia. Leptin, FGF21, AFABP, afamin, and secretagogin were assessed in plasma using the respective Biovendor R&D ELISA kits and adiponectin was determined via the Millipore ELISA kit.

### 2.4. Bioimpedance Analysis

For the BIA, the seca medical Body Composition Analyzer was used after making sure the patients were not using a pacemaker. Prior to measurement, participants were asked to empty their bladder and the analysis began after a resting period in the supine position for a period of ten minutes.

### 2.5. Statistical Analysis

Baseline characteristics are presented via mean and standard deviation and counts and percentages for categorical data. In case of a slight to moderate deviation from the mean, data transformation will be performed. Normality testing will be performed both visually, via histograms, and calculation, via the Shapiro–Wilks test. In the tables, back-transformed values will be presented via mean and range. The between-group differences will be analyzed via a *t*-test and ANOVA or, in case of stark deviation from the mean, via the Wilcoxon rank sum test and Kruskal–Wallis test. *p*-value adjustments were performed via Holm’s method (for overall ANOVAs and Kruskal–Wallis tests) and Shaffer’s correction (for between-group differences assessed via *t*-test and the Wilcoxon rank sum test). Longitudinal analyses were performed via a paired *t*-test and a paired Wilcoxon rank sum test, respectively, and the *p* values were adjusted via Holm’s method. Violin-dot plots for the adipokine analyses are presented in Figures S1 and S2. Linear relationships were assessed via the Pearson correlation coefficient and displayed via a heatmap, and the *p*-values were adjusted after Benjamini Hochberg’s method. The area under the curve calculations was performed via the trapezoidal method. A significance threshold of 0.05 was chosen as the upper limit for all statistical tests.

## 3. Results

Table 1 shows data for age, BMI before pregnancy, and BIA between pregnant women with a history of RYGB, women with obesity, and normal-weight women.

### 3.1. Differences in Body Composition during Pregnancy

Compared to the two control cohorts, women with a history of an RYGB operation had the lowest phase angle (RYGB: 4.84 ± 0.62° vs. obesity: 5.96 ± 0.50° and NW: 5.82 ± 0.57°, adj. *p* < 0.01). Furthermore, women in the RYGB cohort had the lowest cell fraction (RYGB: 45.52 ± 3.79% vs. obesity: 51.69 ± 2.54% and NW: 50.98 ± 2.86%, adj. *p* < 0.01) and the highest ECM/BCM ratio (RYGB: 1.21 ± 0.20 vs. obesity: 0.94 ± 0.10 and NW: 0.97 ± 0.11, adj. *p* < 0.01). Moreover, they had a higher extracellular mass (RYGB: 29.72% (23.99–37.76%) vs. NW: 22.03% (16.71–26.49%), adj. *p* < 0.01) compared to the normal-weight controls. Compared to the controls with obesity, they had a lower basal metabolic rate (RYGB: 1405.63 ± 102.83 cal vs. obesity: 1581.43 ± 75.13 cal, adj. *p* < 0.05) and a body cell mass (RYGB: 24.98 ± 3.26% vs. obesity: 30.54 ± 2.39%, adj. *p* < 0.05). Women with a history of RYGB had lower values than the controls with obesity, yet higher values than the normal-weight controls in the following parameters: BMI (RYGB: 27.2 kg/m^2^ (25–32.6 kg/m^2^) vs. obesity: 37.3 kg/m^2^ (35.9–39 kg/m^2^) and NW: 22.8 kg/m^2^ (19.75–23.25 kg/m^2^), adj. *p* < 0.01), body water (RYGB: 40.17 ± 3.93% vs. obesity: 43.26 ± 3.24% and NW: 33.14 ± 3.24%, adj. *p* < 0.01), lean mass (RYGB: 54.86 ± 5.36% vs. obesity: 59.11 ± 4.43% and NW: 45.27 ± 4.42%, adj. *p* < 0.01) and body fat (RYGB: 30.48% (19.68–53.58%) vs. obesity: 51.64% (39.9–70.47%) and NW: 23.82% (16.41–35.73%)).

### 3.2. Differences in Adipokine Levels during Pregnancy

**Figure 1:**
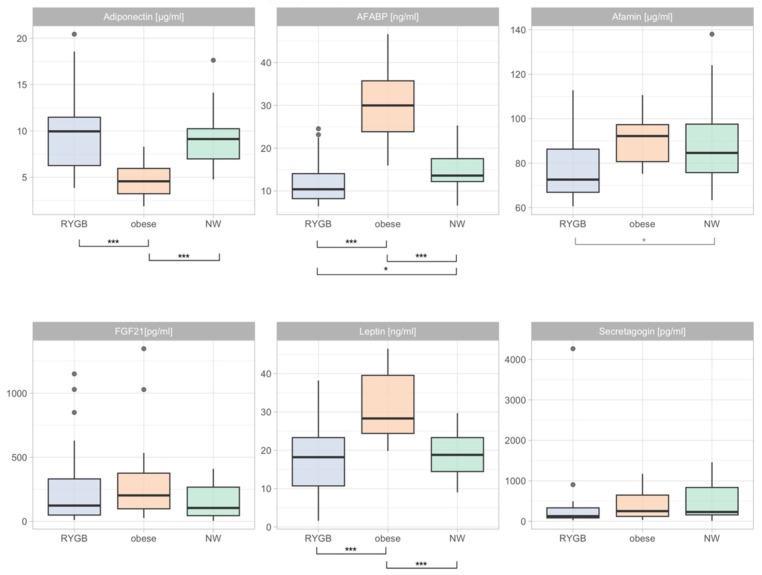
Differences in adiponectin, AFABP, afamin, FGF21, leptin, and secretagogin between women with a history of RYGB, women with obesity, and normal-weight controls. Mean, standard deviation (adiponectin and leptin), and range (afamin, FGF21, and secretagogin) were added to the plot. *p*-values in black indicate significance after Holm’s correction for ANOVA, and the *p*-values in grey indicate unadjusted significant differences. *p*-values between the individual groups were adjusted via Shaffer’s method. RYGB = Roux-en-Y gastric bypass, NW = normal weight, AFABP = adipocyte fatty acid binding protein, and FGF21 = fibroblast growth factor 21, *** *p* < 0.001, * *p* < 0.05.

As for the differences in adipokines, the between-group analyses revealed that, as opposed to post-partum (Table S1), the differences were more pronounced during pregnancies between women with a history of an RYGB operation, women with obesity, and normal-weight controls. Figure 1 depicts these differences.

During pregnancy, significant between-group differences could be seen for leptin, adiponectin, and AFABP, but not FGF21 or secretagogin. Pregnant women with a history of RYGB had significantly lower AFABP (RYGB: 11.19 ng/mL (6.43–24.55 ng/mL) vs. obesity: 28.91 ng/mL (15.96–46.56 ng/mL), adj. *p* < 0.01) and leptin (RYGB: 17.74 ± 10.08 ng/mL vs. obesity: 31.28 ± 8.86 ng/mL, adj. *p* < 0.01) levels in comparison to pregnant women with obesity. At the same time, they had significantly higher adiponectin levels (RYGB: 9.33 µg/mL (3.85–20.42 µg/mL) vs. obesity: 4.27 µg/mL (1.87–8.30 µg/mL), adj. *p* < 0.01). Obese women had higher levels of AFABP (NW: 14.13 ng/mL (6.61–25.29) ng/mL vs. OB: 28.91 ng/mL (15.96–46.56) ng/mL, adj. *p* < 0.01) and leptin (NW: 19.14 ± 5.90 ng/mL vs. OB: 31.28 ± 8.86 ng/mL, adj. *p* < 0.01) compared to normal-weight women during pregnancy. Normal-weight women had higher adiponectin levels (NW: 8.71 µg/mL (4.78–17.62 µg/mL) vs. 4.27 µg/mL (1.87–8.30 µg/mL), adj. *p* < 0.01) compared to pregnant women with obesity. Pregnant women with a history of RYGB had lower afamin levels than normal-weight controls, however, the significance was lost after *p*-value adjusting via Holm’s method (Table S1, Figure S1). The exclusion of women with a history of RYGB and a BMI of 30 kg/m^2^ did not change these results.

### 3.3. Longitudinal Changes in Adipokine Levels (Pregnancy vs. Post-Partum)

**Figure 2:**
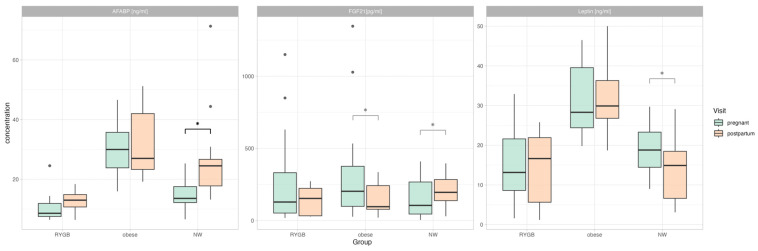
Longitudinal changes of AFABP, FGF21, and leptin in the three cohorts are displayed via boxplots with overlay-dot plots. *p*-values in black indicate significance after Holm’s correction, and *p*-values in grey indicate unadjusted significant differences. RYGB = Roux-en-Y gastric bypass, NW = normal weight, AFABP = adipocyte fatty acid binding protein, FGF21 = fibroblast growth factor 21, and * *p* < 0.05.

Normal-weight women showed an increase in AFABP in the post-partal state (14.13 ng/mL (6.61–25.29 ng/mL) vs. 23.39 ng/mL (13.21–71.29 ng/mL), adj. *p* < 0.05) (Figure 2). FGF21 decreased in the cohort with obesity (196.34 pg/mL (626.73–1345.86 pg/mL) vs. 108.89 pg/mL (20.7–334.97 pg/mL)) and increased in the normal-weight cohort (101.62 pg/mL (4.7–409.26 pg/mL) vs. 180.3 pg/mL (29.99–396.28 pg/mL), however, these significances were lost after correcting for multiple testing. Other than this, there were no significant differences to be seen in the study collective (Table S3). The exclusion of women with a history of RYGB and a BMI of 30 kg/m^2^ did not change these results.

### 3.4. Differences in Adipokine Profiles Post-Partum

Namely during pregnancy, there were significant between-group differences to be seen for leptin, but not AFABP or FGF21 in the post-partal state. Post-partal women with obesity had significantly higher leptin levels than the other two groups (obesity: 32.52 ± 10.57 ng/mL vs. RYGB: 15.32 ± 10.11 ng/mL, adj. *p* < 0.01, NW 14.36 ± 8.59 ng/mL, adj. *p* < 0.01). There was no data available on post-partal adiponectin, afamin, or secretagogin (Table S2, Figure S2). The exclusion of women with a history of RYGB and a BMI of 30 kg/m^2^ did not change these results.

### 3.5. Correlations between Adipokines and Markers of Glucose Homeostasis

**Figure 3:**
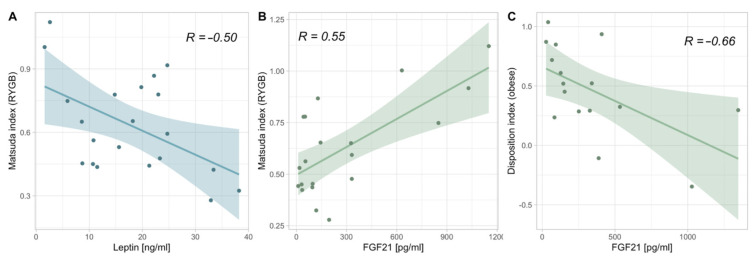
Linear models between (**A**) the Matsuda index and leptin in the RYGB cohort, (**B**) the Matsuda index and FGF21 in the RYGB cohort, and (**C**) the disposition index and FGF21 in the cohort with obesity after adjusting for significance. RYGB = Roux-en-Y gastric bypass, FGF21 = fibroblast growth factor 21.

When assessing the connections between adipokines and the parameters of glucose homeostasis during pregnancy (Figure 3), it became evident that in women with a history of RYGB, FGF21 correlated positively with the Matsuda index (R = 0.55) and leptin correlated negatively with the Matsuda index (R = −0.50). Compared to this, FGF21 correlated negatively with the disposition index in the controls with obesity (R = −0.66). No significant correlations in the normal-weight controls remained after the *p*-value adjustment. Notwithstanding *p*-value correction, there was an association to be seen in the RYGB cohort between the disposition index and leptin (R = −0.50). Likewise, in the cohort with obesity, the initial positive correlation between adiponectin and the Matsuda index (R = 0.52) was lost after the *p*-value adjustment. The exclusion of women with a history of RYGB and a BMI of 30 kg/m^2^ did not change these results.

### 3.6. Correlation Matrix of Adipokines and BIA Parameters

**Figure 4:**
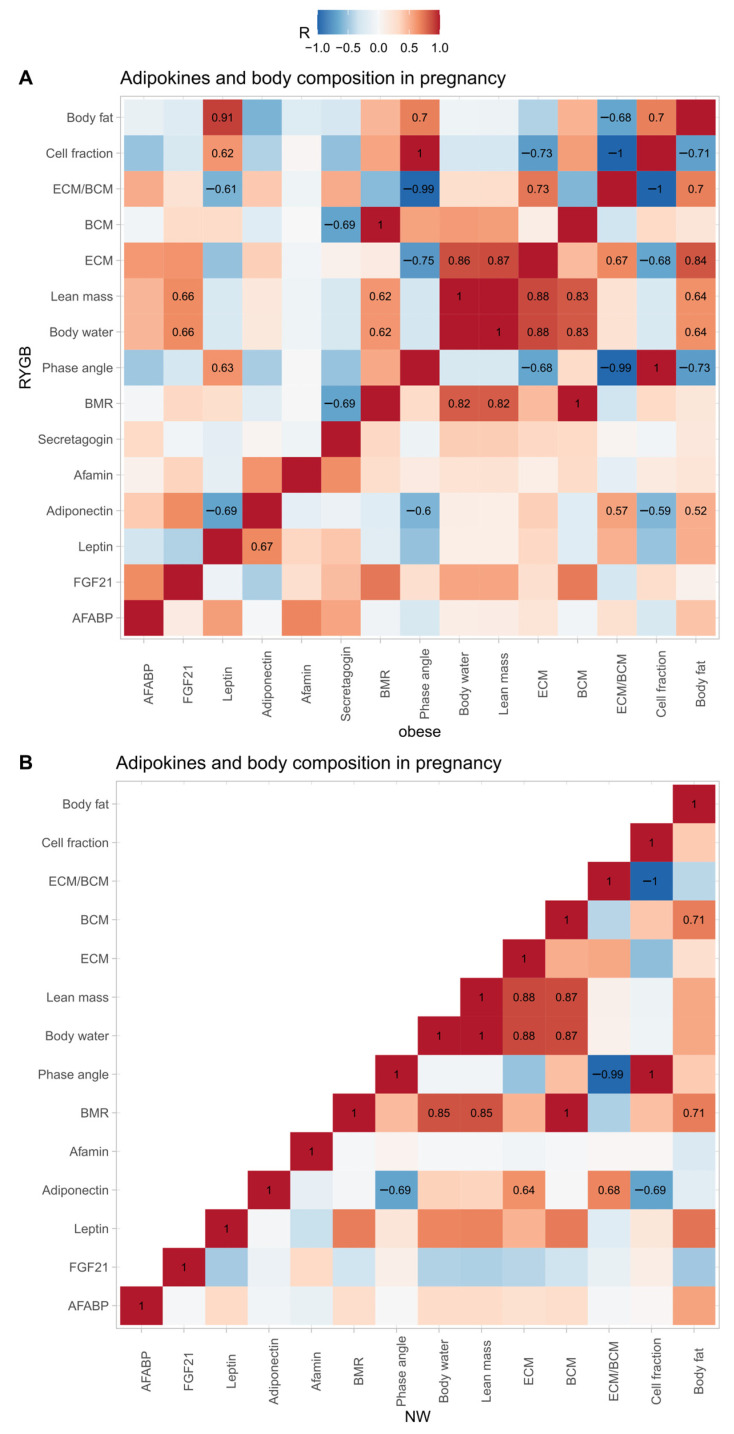
Heatmap of correlations between adipokines and parameters of body composition during pregnancy in women with a history of RYGB ((**A**), upper triangle), obesity ((**A**), lower triangle), and NW controls (**B**). For significant correlations after *p*-value adjustment, the Pearson correlation coefficient was displayed in the respective tiles. RYGB = Roux-en-Y gastric bypass, NW = normal weight, AFABP = adipocyte fatty acid binding protein, FGF21 = fibroblast growth factor 21, BMR = basal metabolic rate, ECM = extracellular mass, and BCM = body cell mass.

Figure 4 displays the correlation matrix of adipokines with parameters of body composition and cellular health. In the cohort of women with a history of RYGB, leptin positively correlated with the phase angle (R = 0.63), BCM cell fraction of the lean mass (R = 0.62), and body fat (0.91), while it negatively correlated with the ratio of extracellular mass (ECM) to BCM (ECM/BCM) (R = −0.61) and adiponectin (R = −0.69). Furthermore, FGF21 correlated positively with body water (0.66) and lean mass (0.66). Of note, before correcting for obesity in the bariatric cohort, there were additional significant correlations (adj. *p* < 0.05) between adipokines and the parameters of body composition to be seen: positive correlations with leptin and basal metabolic rate (BMR; R = 0.54) and body cell mass (BCM; R = 0.55), positive correlations with AFABP and lean mass (R = 0.64), body water (0.64) and ECM (R = 0.57), and a positive correlation of adiponectin with FGF21 (R = 0.72) and afamin (R = 0.77).

Compared to this, in women with obesity, only adiponectin showed a significant association with body composition parameters. In this cohort, adiponectin positively correlated with leptin (R = 0.67), ECM/BCM (R = 0.57), and body fat (R = 0.57), while it correlated negatively with phase angle (R = −0.60) and the BCM cell fraction of the lean mass (R = −0.59).

In the normal-weight controls adiponectin correlated negatively with phase angle (R = −0.69), BCM (R = −0.51) and BCM cell fraction of the lean mass (R = −0.69), while it correlated positively with ECM (R = 0.64) and ECM/BCM (R = 0.68). Of note, before p-value adjustment, there were additional positive correlations to be seen between leptin and basal metabolic rate (R = 0.71), total body water (R = 0.68), lean mass (R = 0.69), body cell mass (R = 0.72) and body fat (R = 0.74).

## 4. Discussion

In this study, we were able to show that there are distinct differences in the adipokine profiles between women with a history of RYGB, women with obesity, and normal-weight women in pregnancy. Concerning body composition, pregnant women with a history of an RYGB take up an intermediary metabolic place between women with obesity and women with normal body weight, except for the phase angle which was the lowest in the RYGB cohort compared to the other cohorts. Furthermore, linear models showed distinct correlations between adipokines and parameters of body composition in women with a history of RYGB, obese and normal-weight pregnant women.

It is interesting that both the body composition and the adipokine profile dynamic between pregnancy and post-partum are significantly different in the RYGB cohort, which seems to be independent of BMI. The substantial weight loss after an RYGB operation leads to a reversal of the disadvantageous metabolic properties associated with obesity [18] and an adjustment of the adipokine secretion pattern [19]. However, it seems as though, at least during pregnancy, not all functions return to the level of normal-weight individuals. As can be seen in the bioelectrical impedance analysis during pregnancy, women with a history of RYGB have the lowest phase angle, a finding which might point toward the poor nutritional status of metabolically active cells [20]. This, in turn, might cause slight malnutrition of the fetus and, thus, an increased risk for premature delivery and small for gestational-age newborns, a finding commonly found in a bariatric collective [21,22].

The negative correlation between adiponectin and metabolic health in the pregnant women with obesity and normal-weight controls seems counterintuitive due to the generally beneficial effect of adiponectin [10]. However, it is possible that adiponectin might in fact be upregulated when cellular health is low, thus resulting in an inverse relationship. Of note, as adiponectin seems to be uncoupled from metabolic health in RYGB women, this might also be an explanation as to why the phase angle is significantly lower in this cohort. The insulin-sensitizing effects of FGF21 [23] correspond well to the findings of this study, i.e., (i) the positive correlation between FGF21 and the Matsuda index in the bariatric cohort, (ii) the counter-regulatory up-regulation in pregnant women with obesity, possibly due to the high risk for GDM and the ensuing surplus of insulin-resistance boosting factors [7], and (iii) the down-regulation in the normal-weight controls, potentially to further enforce the physiological late-gestational insulin resistance [24]. Moreover, the positive correlation between leptin and the phase angle in the bariatric cohort might point towards leptin as an aid to gauge cellular health during pregnancy. The inverse relationship between leptin and the Matsuda index in the pregnant bariatric cohort also corresponds well with this thesis since lower phase angle levels have been associated with insulin resistance [25].

Thus, it is possible that there exists a putative link between adipokines and cellular health during pregnancy after an RYGB operation. Monitoring the profiles of adipokines, such as FGF21 or leptin, in pregnancy post-RYGB operation might be a good indirect indicator for cellular health, glycemic response, and, consequently, detection of individuals at risk of developing GDM. Although, after having undergone a bariatric procedure, the risk of developing GDM is markedly decreased [26], it is still possible that those few women who still develop GDM might go unnoticed due to the lack of routine OGTT testing during pregnancy after RYGB [6,27]. This argument is backed by a study using a continuous glucose monitoring system which found out that pregnant women with an RYGB had similar hyperglycemic times comparable to non-RYGB pregnant women with manifest GDM [28].

In general, adipokines seem to be differently related to gestational metabolic health in women with a history of RYGB compared to the normal-weight controls and women with obesity. Furthermore, although not holding up to the *p*-value adjustment, it should be noted that women with a history of RYGB were the only cohort not to show significant alterations in adipokine levels pre- vs. post-partum, indicating a more rigid adipokine profile. Moreover, adipokine physiology during pregnancy also seems to have a cohort-specific phenotype. Thus, a possible long-term effect of obesity on metabo-cellular health, such as epigenetic modification, ought to be discussed. It has been shown that obesity is associated with epigenetic modifications, such as DNA methylation, causing hypermethylation in gene loci which are, among others, responsible for regulating adipogenesis [29]. More specifically, Arner et al. were able to show that in women with obesity, up to 25% of all genes associated with adipogenesis, 19% of genes involved in insulin signaling, and 41% of genes involved in fatty acid synthesis had significantly differentially methylated DNA sites, resulting in a mostly repressed gene expression pattern [30]. Thus, it might be possible that even after substantial weight loss, these epigenetic modifications persist, leading to distinct adipokine physiology.

Regarding limitations of this study, as this is a post hoc analysis of a prospective clinical study, it is possible that the analyses performed in this study are underpowered and the *p*-value adjustment methods were too conservative in this setting, thus, more subtle effects might go unnoticed. Moreover, due to the limited sample size, more advanced statistical analyses, such as regression models, were not feasible. Additionally, some women in the RYGB cohort presented with a BMI of 30 kg/m^2^ or above and the BMI data points are missing in two cases. Although this did not change the results in the majority of analyses, it is still an important issue to be kept in mind when considering the data. Furthermore, many women refrained from participating in the follow-up post-partal visit, especially in the cohort with obesity. Therefore, the longitudinal analyses might not represent the study collective fully.

## 5. Conclusions

There are significant differences in body composition as well as concentrations and function of adipokines, such as leptin, adiponectin, and FGF21, between pregnant women with a history of RYGB, women with obesity, and normal-weight controls. Furthermore, as there are distinct relationships between adipokines and parameters of glucose metabolism, such as the Matsuda index and the disposition index, as well as parameters of body composition in the respective cohorts to be seen, adipokines might play an important role in the homeostasis of metabo-cellular health during pregnancy. Crucially, adipokines are not related to the modulation of hypoglycemia risk in women with a history of an RYGB operation. Thus, this study highlights the possible usefulness of indirect cellular-metabolic screening in a highly vulnerable cohort exempt from routine OGTT testing for dysglycemia during pregnancy.

## Figures and Tables

**Table 1:** Baseline characteristics and comparison of bioimpedance parameters between pregnant women with a history of RYGB, women with obesity, and normal-weight controls.

	RYGB	Obesity	NW	*p*-Value	Adj. *p* Value
Age [years]	n = 25	n = 19	n = 19	0.79 ^1^	1 ^1^
Mean (SD)	31.44 (6.98)	31.37 (4.89)	30.26 (5.60)		
BMI [kg/m^2^] *	n = 23	n = 19	n = 19	<0.001 ^2^	<0.001 ^2^
Median (Q1–Q3)	27.2 (25–32.6) ^b d^	37.3 (35.9–39) ^b e^	22.8 (19.75–23.25) ^d e^		
BMR [cal]	n = 16	n = 14	n = 15	<0.001 ^1^	<0.001 ^1^
Mean (SD)	1405.63 (102.83) ^b^	1581.43 (75.13) ^b e^	1346 (78.27) ^e^		
Phase angle [°]	n = 16	n = 14	n = 15	<0.001 ^1^	<0.001 ^1^
Mean (SD)	4.84 (0.62) ^b d^	5.96 (0.5) ^b^	5.82 (0.57) ^d^		
Body water [%]	n = 16	n = 14	n = 15	<0.001 ^1^	<0.001 ^1^
Mean (SD)	40.17 (3.93) ^a d^	43.26 (3.24) ^a e^	33.14 (3.24) ^d e^		
Lean mass [%]	n = 16	n = 14	n = 15	<0.001 ^1^	<0.001 ^1^
Mean (SD)	54.86 (5.36) ^a d^	59.11 (4.43) ^a e^	45.27 (4.42) ^d e^		
ECM [%]	n = 16	n = 14	n = 15	<0.001 ^3^	<0.001 ^3^
Mean (range)	29.72 (23.99–37.76) ^d^	28.44 (24.38–34.67) ^e^	22.03 (16.71–26.49) ^b e^		
BCM [%]	n = 16	n = 14	n = 15	<0.001 ^1^	<0.001 ^1^
Mean (SD)	24.98 (3.26) ^b^	30.54 (2.39) ^b e^	23.07 (2.48) ^e^		
ECM/BCM	n = 16	n = 14	n = 15	<0.001 ^1^	<0.001 ^1^
Mean (SD)	1.21 (0.2) ^b d^	0.94 (0.1) ^b^	0.97 (0.11) ^d^		
Cell fraction [%]	n = 16	n = 14	n = 15	<0.001 ^1^	<0.001 ^1^
Mean (SD)	45.52 (3.79) ^b d^	51.69 (2.54) ^b^	50.98 (2.86) ^d^		
Body fat [%]	n = 16	n = 14	n = 15	<0.001 ^3^	<0.001 ^3^
Mean (range)	30.48 (19.68–53.58) ^b c^	51.64 (39.9–70.47) ^b e^	23.82 (16.41–35.73) ^c e^		

* BMI before pregnancy. RYGB = Roux-en-Y gastric bypass, NW = normal weight, ECM = extracellular mass, BCM = body cell mass, BMR = basal metabolic rate, ^a^ = *p*-value < 0.05 between RYGB and women with obesity, ^b^ = *p*-value < 0.01 between RYGB and women with obesity, ^c^ = *p*-value < 0.05 between RYGB and normal-weight women, ^d^ = *p*-value < 0.01 between RYGB and normal-weight women, ^e^ = *p*-value < 0.01 between women with obesity and normal-weight women, ^1^ = ANOVA, ^2^ = Kruskal–Wallis test, ^3^ = ANOVA (after log10 transformation).

## Data Availability

The data cannot be made available to the public.

## Supplementary Materials

The following supporting information can be downloaded at: https://www.mdpi.com/article/10.3390/nu15112498/s1, Figure S1: Violin-dot-plot for the comparisons in Figure 1/Table S1; Figure S2: Violin-dot-plot for the comparisons in Figure 2/Table S2; Table S1: Differences between adipokines during pregnancy; Table S2: Differences between adipokines postpartum; Table S3: Longitudinal changes of adipokines (pregnancy vs. postpartum).

## References

[1] World Health Organization (WHO) Obesity and Overweight. https://www.who.int/en/news-room/fact-sheets/detail/obesity-and-overweight.

[2] Nguyen N.T., Varela J.E. (2017). Bariatric surgery for obesity and metabolic disorders: State of the art. Nat. Rev. Gastroenterol. Hepatol..

[3] Salehi M., D’Alessio D.A. (2014). Effects of glucagon like peptide-1 to mediate glycemic effects of weight loss surgery. Rev. Endocr. Metab. Disord..

[4] Schauer P.R., Burguera B., Ikramuddin S., Cottam D., Gourash W., Hamad G., Eid G.M., Mattar S., Ramanathan R., Barinas-Mitchel E. (2003). Effect of laparoscopic Roux-en Y gastric bypass on type 2 diabetes mellitus. Ann. Surg..

[5] Lupoli R., Lembo E., Rainone C., Schiavo L., Iannelli A., Di Minno M.N.D., Capaldo B. (2022). Rate of post-bariatric hypoglycemia using continuous glucose monitoring: A meta-analysis of literature studies. Nutr. Metab. Cardiovasc. Dis..

[6] Göbl C.S., Bozkurt L., Tura A., Leutner M., Andrei L., Fahr L., Husslein P., Eppel W., Kautzky-Willer A. (2017). Assessment of glucose regulation in pregnancy after gastric bypass surgery. Diabetologia.

[7] McIntyre H.D., Catalano P., Zhang C., Desoye G., Mathiesen E.R., Damm P. (2019). Gestational diabetes mellitus. Nat. Rev. Dis. Prim..

[8] Plows J.F., Stanley J.L., Baker P.N., Reynolds C.M., Vickers M.H. (2018). The Pathophysiology of Gestational Diabetes Mellitus. Int. J. Mol. Sci..

[9] Salman M.A., El-Ghobary M., Soliman A., El Sherbiny M., Abouelregal T.E., Albitar A., Abdallah A., Mikhail H.M.S., Nafea M.A., Sultan A. (2020). Long-Term Changes in Leptin, Chemerin, and Ghrelin Levels Following Roux-en-Y Gastric Bypass and Laparoscopic Sleeve Gastrectomy. Obes. Surg..

[10] Ouchi N., Parker J.L., Lugus J.J., Walsh K. (2011). Adipokines in inflammation and metabolic disease. Nat. Rev. Immunol..

[11] Recinella L., Orlando G., Ferrante C., Chiavaroli A., Brunetti L., Leone S. (2020). Adipokines: New Potential Therapeutic Target for Obesity and Metabolic, Rheumatic, and Cardiovascular Diseases. Front. Physiol..

[12] Farkhondeh T., Llorens S., Pourbagher-Shahri A.M., Ashrafizadeh M., Talebi M., Shakibaei M., Samarghandian S. (2020). An Overview of the Role of Adipokines in Cardiometabolic Diseases. Molecules.

[13] Lee C.H., Lui D.T.W., Lam K.S.L. (2021). Adipocyte Fatty Acid-Binding Protein, Cardiovascular Diseases and Mortality. Front. Immunol..

[14] Dieplinger H., Dieplinger B. (2015). Afamin—A pleiotropic glycoprotein involved in various disease states. Clin. Chim. Acta.

[15] Maj M., Wagner L., Tretter V. (2019). 20 Years of Secretagogin: Exocytosis and Beyond. Front. Mol. Neurosci..

[16] Deischinger C., Harreiter J., Leitner K., Bancher-Todesca D., Baumgartner-Parzer S., Kautzky-Willer A. (2020). Secretagogin is Related to Insulin Secretion but Unrelated to Gestational Diabetes Mellitus Status in Pregnancy. J. Clin. Med..

[17] Leutner M., Klimek P., Göbl C., Bozkurt L., Harreiter J., Husslein P., Eppel W., Baumgartner-Parzer S., Pacini G., Thurner S. (2019). Glucagon-like peptide 1 (GLP-1) drives postprandial hyperinsulinemic hypoglycemia in pregnant women with a history of Roux-en-Y gastric bypass operation. Metabolism.

[18] Peterli R., Steinert R.E., Woelnerhanssen B., Peters T., Christoffel-Courtin C., Gass M., Kern B., von Fluee M., Beglinger C. (2012). Metabolic and hormonal changes after laparoscopic Roux-en-Y gastric bypass and sleeve gastrectomy: A randomized, prospective trial. Obes. Surg..

[19] Beckman L.M., Beckman T.R., Earthman C.P. (2010). Changes in gastrointestinal hormones and leptin after Roux-en-Y gastric bypass procedure: A review. J. Am. Diet. Assoc..

[20] Norman K., Stobäus N., Pirlich M., Bosy-Westphal A. (2012). Bioelectrical phase angle and impedance vector analysis--clinical relevance and applicability of impedance parameters. Clin. Nutr..

[21] Roos N., Neovius M., Cnattingius S., Trolle Lagerros Y., Sääf M., Granath F., Stephansson O. (2013). Perinatal outcomes after bariatric surgery: Nationwide population based matched cohort study. BMJ.

[22] Hazart J., Le Guennec D., Accoceberry M., Lemery D., Mulliez A., Farigon N., Lahaye C., Miolanne-Debouit M., Boirie Y. (2017). Maternal Nutritional Deficiencies and Small-for-Gestational-Age Neonates at Birth of Women Who Have Undergone Bariatric Surgery. J. Pregnancy.

[23] Flippo K.H., Potthoff M.J. (2021). Metabolic Messengers: FGF21. Nat. Metab..

[24] Lain K.Y., Catalano P.M. (2007). Metabolic changes in pregnancy. Clin. Obstet. Gynecol..

[25] de Luis D.A., Aller R., Romero E., Dueñas A., Perez Castrillon J.L. (2010). Relation of phase angle tertiles with blood adipocytokines levels, insulin resistance and cardiovascular risk factors in obese women patients. Eur. Rev. Med. Pharmacol. Sci..

[26] Yi X.-Y., Li Q.-F., Zhang J., Wang Z.-H. (2015). A meta-analysis of maternal and fetal outcomes of pregnancy after bariatric surgery. Int. J. Gynecol. Obstet..

[27] Harreiter J., Schindler K., Bancher-Todesca D., Göbl C., Langer F., Prager G., Gessl A., Leutner M., Ludvik B., Luger A. (2018). Management of Pregnant Women after Bariatric Surgery. J. Obes..

[28] Bonis C., Lorenzini F., Bertrand M., Parant O., Gourdy P., Vaurs C., Cazals L., Ritz P., Hanaire H. (2016). Glucose Profiles in Pregnant Women After a Gastric Bypass: Findings from Continuous Glucose Monitoring. Obes. Surg..

[29] Agha G., Houseman E.A., Kelsey K.T., Eaton C.B., Buka S.L., Loucks E.B. (2015). Adiposity is associated with DNA methylation profile in adipose tissue. Int. J. Epidemiol..

[30] Arner P., Sinha I., Thorell A., Rydén M., Dahlman-Wright K., Dahlman I. (2015). The epigenetic signature of subcutaneous fat cells is linked to altered expression of genes implicated in lipid metabolism in obese women. Clin. Epigenet..

