# Supplementary material for: The Interplay of Adipokines, Body Composition and Glucose Homeostasis in Pregnant Women with a History of RYGB Operation
Source: Nutrients. 2023 May 27;15(11):2498. doi: 10.3390/nu15112498 (PMC10255888; doi:10.3390/nu15112498)
Supplement: Supplementary file 1 [file nutrients-15-02498-s001.zip › nutrients-2355555-supplementary.pdf]

The interplay of adipokines, body composition and glucose homeostasis in pregnant women with a history of RYGB operation

Luise Bellach MD [1], Liliana-Imi Gard [1], Simon D. Lindner MSc [2,3], Sabina Baumgartner-Parzer PhD [1], Peter Klimek PhD [2,3], Alexandra Kautzky-Willer MD [1], Michael Leutner MD [1]

Corresponding: Michael Leutner MD [1]

Affiliation:

[1] Clinical Division of Endocrinology and Metabolism, Department of Internal Medicine III, Medical University of Vienna, Austria, Spitalgasse 23, A-1090 Vienna, Austria

[2] Section for Science of Complex Systems, CeMSIIS, Medical University of Vienna, Spitalgasse 23, A-1090 Vienna, Austria

[3] Complexity Science Hub Vienna, Josefstädter Strasse 39, A-1080 Vienna, Austria

Supplementary materials

<u>Table of contents</u>

Table S1: Differences between adipokines during pregnancy 2

Figure S1: Violin-dot-plot for the comparisons in Figure 1 / Table S1 2

Table S2: Differences between adipokines postpartum 3

Figure S2: Violin-dot-plot for the comparisons in Figure 2 / Table S2 3

Table S3: Longitudinal changes of adipokines (pregnancy vs. postpartum 4

Table S1: Differences between adipokines during pregnancy

	RYGB (N=25)	obesity (N=19)	NW (N=19)	p-value	Adj. p value
**Leptin [ng/ml]**					
**n**	21	18	19	**< 0.001** [1]	**< 0.001** [1]
**Mean (SD)**	17.74 (10.08) [b]	31.28 (8.86) [b f]	19.14 (5.90) [f]		
**Adiponectin [µg/ml]**					
**n**	21	18	19	**< 0.001** [2]	**< 0.001** [2]
**Mean (range)**	9.33 (3.85 – 20.42) [b]	4.27 (1.87 – 8.30) [b f]	8.71 (4.78 – 17.62) [f]		
**AFABP [ng/ml]**					
**n**	21	18	19	**< 0.001** [2]	**< 0.001** [2]
**Mean (range)**	11,19 [b c] (6,43 - 24,55)	28,91 [b f] (15,96 - 46,56)	14,13 [c f] (6,61 - 25,29)		
**FGF21 [pg/ml]**					
**n**	21	18	17	0.27 [2]	0.27 [2]
**Mean (range)**	4.88 (1.35)	5.28 (1.05)	4.62 (1.15)		
**Afamin [µg/ml]**					
**n**	21	18	19	**0.03** [2]	0.20 [2]
**Mean (range)**	77.98 (60.53 - 112.72) [c]	90.16 (75.16 - 110.66)	88.72 (63.39 - 138.04) [c]		
**Secretagogin [pg/ml]**					
**n**	21	15	19	0.42 [2]	1 [2]
**Mean (range)**	162.55 (25.88 - 4265.8)	234.42 (33.42 - 1172.2)	267.3 (12.91 - 1458.81)		

Table S 1: Comparison of adipokines between pregnant women with a history of RYGB, women with obesity and normal-weight controls. RYGB = Roux-En-Y gastric bypass, NW = normal weight, AFABP = adipocyte fatty acid binding protein, FGF21 = fibroblast growth factor 21, a = p-value < 0.05 between RYGB and women with obesity, b = p-value < 0.01 between RYGB and women with obesity, c = p-value < 0.05 between RYGB and normal-weight women, d = p-value < 0.01 between RYGB and normal-weight women; e = p-value < 0.05 between women with obesity and normal-weight women, f = p-value < 0.01 between women with obesity and normal-weight women, 1 = ANOVA, 2 = ANOVA (after log10 transformation)

Figure S1: Violin-dot-plot for the comparisons in Figure 1 / Table S1

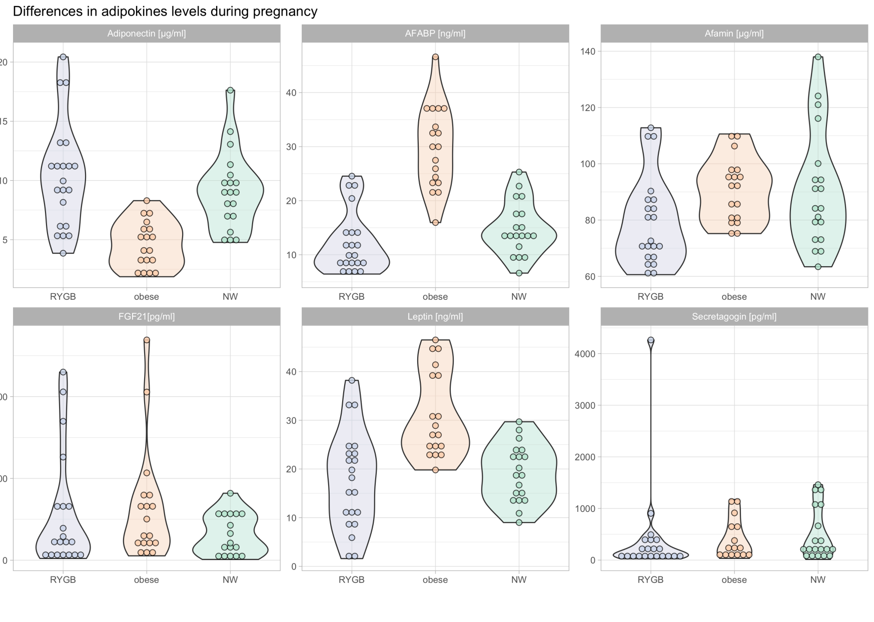

Figure S 1: Violin-dot-plot for the adipokines and group comparisons of Figure 1 and Table S1

Table S2: Differences between adipokines postpartum

	RYGB (N=25)	obesity (N=19)	NW (N=19)	p value	Adj. p value
**Leptin [ng/ml]**					
**n**	12	9	14	**< 0.001** [1]	**< 0.01** [1]
**Mean (SD)**	15.32 (10.11) [b]	32.52 (10.57) [b f]	14.36 (8.59) [f]		
**AFABP [ng/ml]**					
**n**	13	9	14	**0.014** [2]	0.24 [2]
**Mean (SD)**	2.76 (0.54) [a c]	3.38 (0.36) [a]	3.15 (0.47) [c]		
**FGF21 [pg/ml]**					
**n**	13	9	5	0.589 [2]	1 [2]
**Mean (SD)**	5.06 (1.60)	4.69 (0.99)	5.20 (0.65)		

Table S 2: Comparison of adipokines between post-partum women with a history of RYGB, women with obesity and normal-weight controls. RYGB = Roux-En-Y gastric bypass, NW = normal weight, AFABP = adipocyte fatty acid binding protein, FGF21 = fibroblast growth factor 21, a = p-value < 0.05 between RYGB and women with obesity, b = p-value < 0.01 between RYGB and women with obesity, c = p-value < 0.05 between RYGB and normal-weight women, d = p-value < 0.01 between RYGB and normal-weight women; e = p-value < 0.05 between women with obesity and normal-weight women, f = p-value < 0.01 between women with obesity and normal-weight women, 1 = ANOVA, 2 = ANOVA (after log10 correction)

Figure S2: Violin-dot-plot for the comparisons in Figure 2 / Table S2

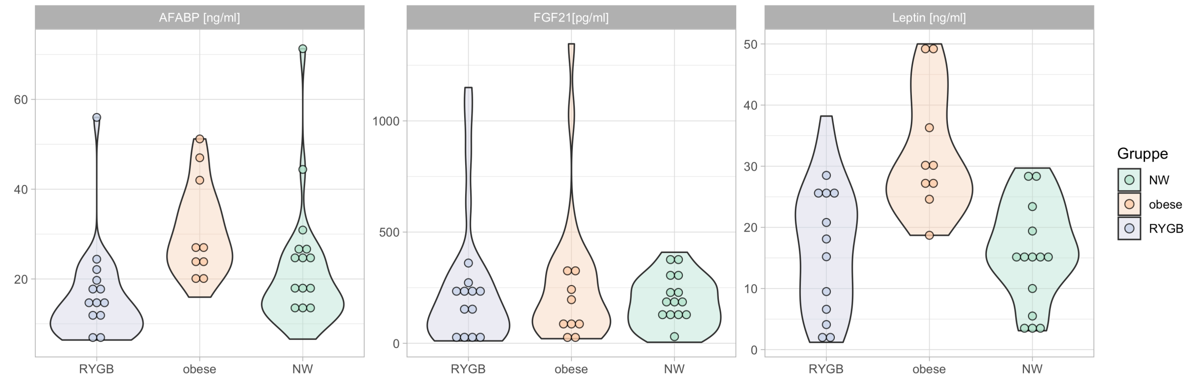

Figure S 2: Violin-dot-plot for the adipokines and group comparisons of Figure 1 and Table S1

Table S3: Longitudinal changes of adipokines (pregnancy vs. postpartum)

		gestational	post-partum	p-value	Adj. p value
**RYGB**					
	**Leptin [ng/ml]**				
	**n**	21	12	0.57 [1]	1 [1]
	**Mean (SD)**	17.74 (10.08)	15.32 (10.11)		
	**AFABP [ng/ml]**				
	**n**	21	13	0.32 [2]	1 [2]
	**Mean (range)**	11.19 (6.43 - 24.55)	15.85 (6.4 - 55.98)		
	**FGF21 [pg/ml]**				
	**n**	20	13	0.63 [2]	1 [2]
	**Mean (range)**	131.22 (10.69 - 1150.8)	157.04 (20.18 - 9354.06)		
**obesity**					
	**Leptin [ng/ml]**				
	**n**	18	9	0.32 [1]	1 [1]
	**Mean (SD)**	31.28 (8.86)	32.52 (10.57)		
	**AFABP [ng/ml]**				
	**n**	18	9	0.92 [2]	0.92 [2]
	**Mean (SD)**	28.91 (15.96 - 46.56)	29.51 (19.19 - 51.17)		
	**FGF21 [pg/ml]**				
	**n**	18	9	**0.03 [2]**	0.28 [2]
	**Mean (SD)**	196.34 (26.73 - 1345.86)	108.89 (20.7 - 334.97)		
**NW**					
	**Leptin [ng/ml]**				
	**n**	19	14	**0.02 [1]**	0.22 [1]
	**Mean (SD)**	19.14 (5.90)	14.36 (8.59)		
	**AFABP [ng/ml]**				
	**n**	19	14	**0.002 [2]**	**0.024 [2]**
	**Mean (SD)**	14.13 (6.61 - 25.29)	23.39 (13.21 - 71.29)		
	**FGF21 [pg/ml]**				
	**n**	17	14	**0.03 [2]**	0.28 [2]
	**Mean (SD)**	101.62 (4.7 - 409.26)	180.3 (29.99 - 396.28)		

Table S 3: Comparison of adipokines between gestational versus post-partal period in women with a history of RYGB, women with obesity and normal-weight controls. RYGB = Roux-En-Y gastric bypass, NW = normal weight, AFABP = adipocyte fatty acid binding protein, FGF21 = fibroblast growth factor 21, 1 = Student's paired t test, 2 = Student's paired t test (after log10 transformation)
